# Level and Contamination Assessment of Soil along an Expressway in an Ecologically Valuable Area in Central Poland

**DOI:** 10.3390/ijerph121013372

**Published:** 2015-10-23

**Authors:** Maja Radziemska, Joanna Fronczyk

**Affiliations:** 1 Department of Environmental Improvement, Faculty of Civil and Environmental Engineering, Warsaw University of Life Sciences, Nowoursynowska 159, 02-776 Warsaw, Poland; 2 Department of Geotechnical Engineering, Faculty of Civil and Environmental Engineering, Warsaw University of Life Sciences, Nowoursynowska 159, 02-776 Warsaw, Poland; E-Mail: joanna_fronczyk@sggw.pl

**Keywords:** roadside hazards, heavy metals, expressways and roads, protected areas, pollution estimation

## Abstract

Express roads are a potential source of heavy metal contamination in the surrounding environment. The Warsaw Expressway (E30) is one of the busiest roads in the capital of Poland and cuts through the ecologically valuable area (Mazowiecki Natural Landscape Park). Soil samples were collected at distances of 0.5, 4.5 and 25 m from the expressway. The concentrations of cadmium (Cd), copper (Cu), nickel (Ni), lead (Pb), and zinc (Zn) were determined in the soils by the flame atomic absorption spectrometry method (FAAS). Soils located in the direct proximity of the analyzed stretch of road were found to have the highest values of pH and electrical conductivity (EC), which decreased along with an increase in the distance from the expressway. The contents of Cd, Cu and Zn were found to be higher than Polish national averages, whereas the average values of Ni and Pb were not exceeded. The pollution level was estimated based on the geo-accumulation index (*I_geo_*), and the pollution index (*PI*). The results of *I_geo_* and *PI* indexes revealed the following orders: Cu < Zn < Ni < Cd < Pb and Cu < Ni < Cd < Zn < Pb, and comparison with geochemical background values showed higher concentration of zinc, lead and cadmium.

## 1. Introduction

The natural environment is a system of interconnected elements, with anthropogenic activities constituting the main source of its pollution, especially that affecting the soil and water. Using natural resources inadequately leads to an offset of the chemical balance. The excessive accumulation of heavy metals in the lithosphere, hydrosphere and atmosphere is a serious threat to living organisms, including humans [1,2]. The development of civilization and the industrialization that accompanied it led to the severe pollution of the natural environment in vast areas of many countries. In addition to industry and farming, the main source of contamination of the environment with heavy metals is the transportation infrastructure, which includes roads, bridges and overpasses. International research centers have documented the negative influence of roads on the physicochemical properties of water and soil [3,4,5,6,7]. On the other hand, Perez *et al.* [8] could not find a direct correlation between traffic intensity and metal concentrations in roadside soil. The mains source of heavy metal contamination in the proximity of roads are tire and brake abrasion, combustion exhaust, pavement wear and the application of road salt in the winter period [9,10]. These substances migrate to the ground and groundwater along with runoff from the surface of the roads, which is regarded as one of the sources significantly affecting the quality of surface- and groundwater [11,12]. The content of pollutants in runoff water infiltrating the soil is dependent on many factors, including the intensity of traffic, type of road and condition of the road surface. The increased concentration of heavy metals over a long period of time can be potentially associated with their accumulation in soil and pose a risk to the proper functioning of the water ecosystem. Dust derived from motor vehicles contains particles of abradants which are worn down together with the exploitation of the vehicle (brake and clutch pad linings, tire materials and road surfaces). The heavy metal containing dust also settles on nearby plants and the soil, leading to the increase in the natural values of these element, which in turn impedes the course of the vegetation process of plants and destroys the ecological and esthetic values of the greenery [13,14].

In order to eliminate the negative effects of roads on the quality of surface- and groundwater, engineering solutions such as separators or settlement and infiltration tanks are applied [3]. The processes responsible for the transportation of pollutants in the areas of roads are analogical to the processes taking place in areas affected by other anthropogenic sources. The main and most important heavy metals include copper, cadmium, lead, zinc and nickel [15,16]. Heavy metals are characterized by a low ability to migrate, which is connected with forming not easily soluble compounds which easily accumulate in soil [17]. Their content in soil is connected with the distance from the transportation route, intensity of traffic, structure of the landforms and land use. The surface layer of soil is characterized by the highest heavy metal content [18].

The article focuses on the assessing the degree to which an expressway located in areas of ecological importance influences selected parameters of the soil (pH, EC) as well as the content of selected heavy metals (Zn, Pb, Ni, Cd, Cu) in the soil profile at five depths and their effect at various distances from the road.

## 2. Material and Methods

### 2.1. Study Site

**Figure 1:**
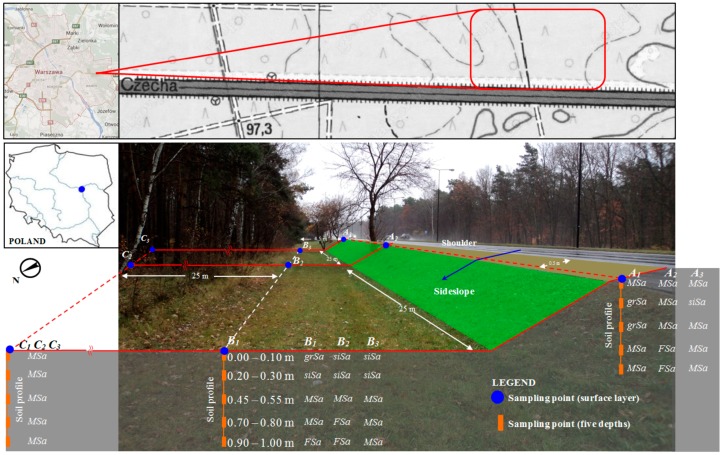
The study area and the soil sampling locations. MSa: medium sand; grSa: sand with gravel; siSa: silty sand; FSa: fine sand.

The study site was a stretch of a national road regarded as the most important European transportation route on the east-west axis and the main route taken by large trucks transporting goods from Western Europe to Russia and Belarus. The sampling locations (Figure 1) were located along expressway E30, which cuts through the ecologically valuable terrain of the Mazowiecki Natural Landscape Park, Central Poland (52°3′32″ north altitude and 21°10′5″ east longitude). The protected area was established in 1986 and covers an area of 143.7 km^2^. The expressway has two lays going in each direction, each of which is 4 m in width, and the surfaces are asphalt concrete pavement, average daily traffic (veh/day) 53,998. The speed of the vehicles was 50–90 km·h^−1^. Most of the vehicles running on this road use fossil fuel *i.e.*, diesel, gasoline, and natural gas. The road was constructed on the embankment of 1.4 m height filled with medium sand grained material. Material extracted from Section B and C (medium and fine sands) was natural soil, while material in embankment (Section A) was brought from other area and filled with compaction. On the analyzed stretch of road, runoff directly infiltrates into the adjacent soil. The surrounding land use is a mixed pine-oak forest, which starts approximately 5 m from the edge of the road. Poland is located in the temperate warm transitional zone, forming under the influence of different air masses clashing over its territory. The average annual precipitation in Poland is around 628 mm, but in the mountainous areas it exceeds 1100 to 1400 mm. Most rainy season falls between in May, June, July and August.

### 2.2. Soil Sample Collection

Soil samples were collected in two directions, vertically and horizontally, along a 50 m stretch of road. The soil samples were taken in a 50 m stretch from the top of the embankment (0.5 m from the edge of the road) and at the bottom (4.5 m and 25 m from the edge of the road), from five different depths. From each section (A, B and C), fifteen samples were taken from each sampling depth, *i.e.*, 0.00-0.10 m, 0.20-0.30 m, 0.45-0.55 m, 0.70-0.80 m and 0.90-1.00 m. Soil collection from the 0.00–0.10 m layer was carried out with Egner’s soil sampler, whereas soil samples from the deeper layers, *i.e.*, 0.20–1.00 m, were collected using a soil auger. The measurement profiles were chosen in such a way as to guarantee the high reproducibility of results, and were located on a single side of the road. Soil samples from five locations were mixed into a single representative sample. Prior to chemical analyses, soil samples were prepared by being dried at room temperature and sifted through a 1 mm polyethylene sieve to remove stones, coarse materials, and other debris, and then stored in polyethylene bottles.

### 2.3. Chemical and Physical Analysis

The following analyses were performed on the soil samples: pH (exchangeable acidity)—determined by means of the potentiometric method using an aquatic solution of KCl at a concentration of 1 M·KCl·dm^−3^ with a glass electrode and a Handylab pH/LF 12 pH meter (Schott, Germany), electrical conductivity (EC)—Measured with a Handylab pH/LF 12 conductometer (Schott, Germany), in a 1:2 soil/deionized water suspension (w/v). The metals selected for the study were distinguished by diverse degrees of anthropogenic origin. The total contents of zinc (Zn), lead (Pb), nickel (Ni), cadmium (Cd) and copper (Cu), were determined in extracts obtained upon mineralization in nitric acid (HNO_3_ p.a.) with a concentration of 1.40 g·cm^−1^ and 30% H_2_O_2_ in a MARS 5 microwave oven (CEM Corporation, USA), in HP500 teflon vessels (the parameters of the process, *i.e.*, weight of analytical samples, volume of nitric acid, and temperature of the mineralization process complied with the US-EPA3051 Protocol) [19]. Total concentrations of the five analyzed heavy metals were determined by means of the flame atomic absorption spectrometry (FAAS) method on a SpectrAA 240FS spectrometer (VARIAN, Australia) in an air-acetylene flame, using a Sample Introduction Pump System (SIPS). The analysis of heavy metal content was performed in triplicate.

All standards, reagent solutions and samples were kept in polyethylene containers. All plastic and glassware was washed at least three times with de-ionized water, soaked in HNO_3_, again rinsed in de-ionized water and finally, dried in an oven. Standard metal solutions (1000 mg·L^−1^) were purchased from Merck (Darmstadt, Germany). All reagents were of analytical reagent grade unless otherwise stated. Double deionized water (Milli-Q Millipore, USA) was used for all dilutions. The solution of each sample was cooled and filtered on Whatman prewashed filter paper.

Particle size distribution of collected soils was determined using ISO/TS 17892-4 [20].

### 2.4. Contamination Assessment Methods

The geo-accumulation index (*I_geo_*) [21] and pollution index (*PI*) [22] were used to assess the contamination degrees of heavy metals for the study region. These indexes were widely used in trace metal studies of soils [23]. The *I_geo_* was calculated by Equation (1):

(1) $$I_{geo}=log_{2}\left[\frac{C_{n}}{1.5B_{n}}\right]$$

where *C_n_* is the measured concentration of the element *n*, and *B_n_* is the geochemical background value of the element; and the PI using Equation (2):

(2) $$PI=\frac{C_{n}}{B_{n}}$$

The *B_n_* values for Zn, Pb, Cd, Ni and Cu were based on the Geochemical atlas of Poland, Warsaw [24], which took the following values: 25 mg·Zn·kg^−1^; 12.5 mg·Pb·kg^−1^; 0.5 mg·Cd·kg^−1^; 5.0 mg·Ni·kg^−1^; 10 mg·Cu·kg^−1^. The classification of soil contamination using *I_geo_* and *PI* indexes is presented in Table 1.

**Table 1:** Soil pollution degrees based on *I_geo_* and *PI*.

*I_geo_* [21]	*I_geo_* Class	Pollution Category	*PI* [22]	Pollution Category
*I_geo_* ≤ 0	0	Uncontaminated	≤1	low
0 < *I_geo_* ≤ 1	1	Uncontaminated to moderately contaminated	1–3	middle
1 < *I_geo_* ≤ 2	2	Moderately contaminated	>3	high
2 < *I_geo_* ≤ 3	3	Moderately to strongly contaminated		
3 < *I_geo_* ≤ 4	4	Strongly contaminated		
4 < *I_geo_* ≤ 5	5	Strongly to extremely contaminated		
*I_geo_* ≥ 5	6	Extremely contaminated		

### 2.5. Statistical Treatment

The results were processed statistically using a one-way analysis of variance (ANOVA) from Statistica [25], calculating mean values and standard deviation. Pearson’s simple correlation coefficient (*r*) was also calculated between the heavy metal content indicated in the soil and the distance from the expressway, with the level of significance set at *p* < 0.05 and *p* < 0.01.

## 3. Results and Discussions

**Figure 2:**
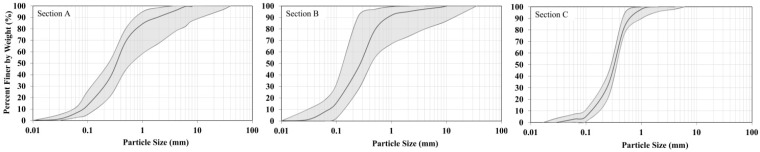
Ranges of particle size variability in the analyzed Sections (**A**–**C**).

**Figure 3:**
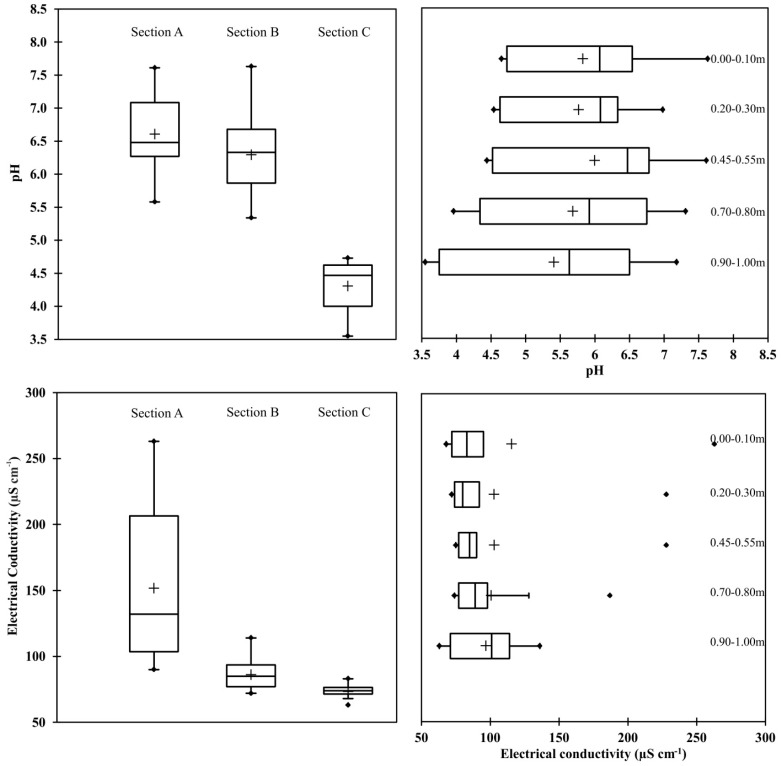
Boxplots showing the median, quartiles, and minimum and maximum values of the pH and electrical conductivity (EC) with the depth and distance values from the road.

The particle size distribution of soils collected from different distances from the road (sections) are presented in Figure 2. The chemical composition of the soil depended on the distance from the road, depth and sampling location (Figure 2 and Figure 3). The physical, chemical, and biological properties of soil are directly dependant on its pH value [26]. The soil pH near roads is influenced strongly by traffic activities. When determining the dynamics of changes in pH at the five analyzed depths of soil, samples taken directly next to the road at three sampling locations were characterized by higher values of this parameter (Figure 3). Decreasing values of pH were recorded along with an increase in the distance from the expressway. Studies conducted by Lee *et al.* [27] confirmed a higher pH of soil in the direct proximity of a road. Soil samples collected at sampling locations nearest to the road (0.5 m) in the present study were characterized by a pH ranging from 5.58–7.18, with an average value of 6.58. Numerous factors may have led to the differences in the values of pH determined in the soil samples collected from the analyzed stretch of the transport route. It is possible that the presence of a forest community accounted for the reported soil acidity, due to the decomposition processes of organic material which occurs there and produces organic acids. Road abrasion, which is transferred to the soil adjacent to the road, changes the pH value over time to neutral or even above neutral. The pH value of soil at a distance of 25 m from the road was significantly correlated (*r* = 0.927) with increasing depth.

Values of electric conductivity (EC) determined in the soil samples depended on the sampling location as well as the distances at which they were collected. Soil collected at a distance of 0.5 m was found to have the highest values of this parameter (163 µS·cm^−1^), with samples taken 25 m from the road characterized by the lowest (63 µS·cm^−1^); this can be due the application of salt in winter to prevent ice. At 4.5 m from the analyzed stretch of road, a significant negative correlation (*r* = −0.788) was observed between the values of electrical conductivity (EC) and the soil sample collection depth.

### 3.1. Concentration of Heavy Metals in Soil

**Figure 4:**
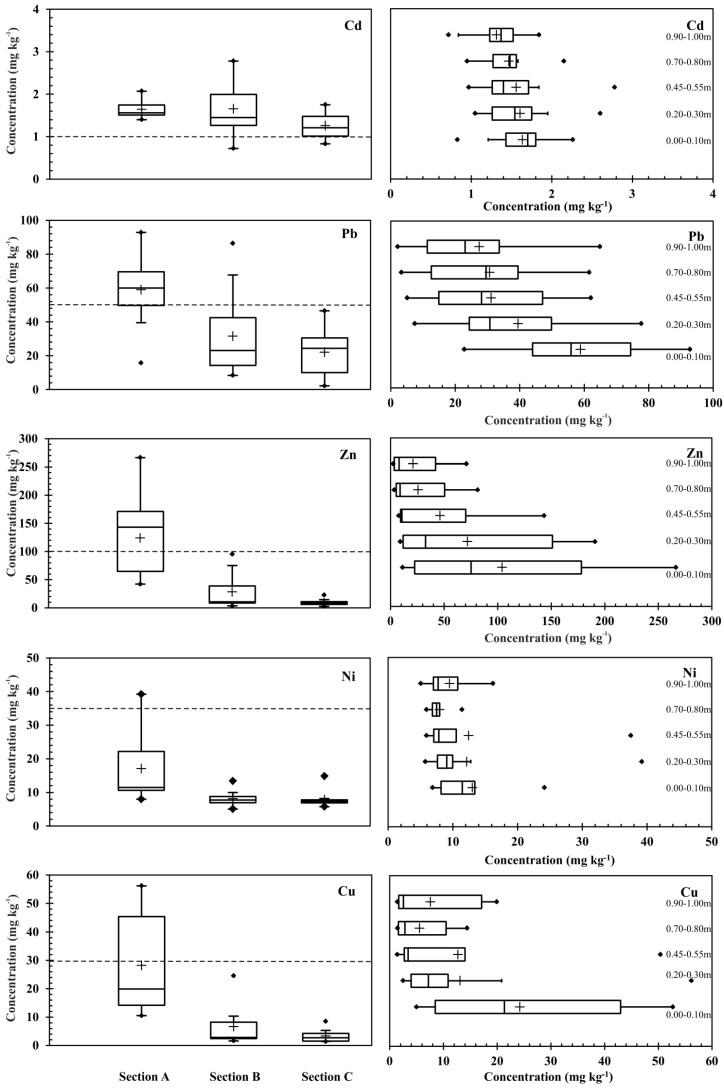
Boxplots showing the median, quartiles, and minimum and maximum values of the heavy metals concentrations with the depth and distance from the road. Dotted horizontal line represent heavy metal level in protected areas according Polish law.

The chemical properties of the soil (minimum, maximum, and mean values, as well as standard deviation) at increasing distances from the expressway (E30) have been presented in Figure 4. The contents of the analyzed heavy metals in the soil samples depended on the site from which the samples were taken, as well as on the distance from the expressway. The data from the surface layers (0.0 m and 0.25 m) represented the effect of the parent-material and traffic activity, while from the deeper layers (0.75 m and 1.0 m) of each sampling points represented the effect of parent-material. The average metal concentrations at all sampling sites can be ranked as follows: Zn > Pb > Cu > Ni > Cd. Soils collected nearest to the road (0.5 m) were characterized by the highest contents of the analyzed heavy metals, the concentration of which decreased the further the soil was from the expressway. The decrease of heavy metal concentrations with distance to the road is well documented by many authors e.g., [28,29].

Cadmium (Cd) is a very toxic element, with its presence near roads being attributed to dust from the combustion of petrol, in brake linings and is also present in the rubber used for tire production [30,31]. The concentration of Cd in the soil samples from locations near the expressway varied from 1.43 to 2.07 mg·kg^−1^, with an average value of 1.62 mg·kg^−1^ of soil. The Regulation of the Polish Minister of the Environment [32] specifies the permissible amounts of cadmium as 1 mg·kg^−1^ of soil (protected areas), and 4 mg·kg^−1^ of soil (unprotected areas, including farmland), up to 15 mg·kg^−1^ of soil (land under transportation routes). The concentrations of cadmium were found to be lower when compared to other metals determined in the analyzed soil samples. This corresponds with the results of other studies, such as those conducted by Al-Khashman [33]. Soils in uncontaminated regions of Poland contain 0.1–0.6 mg·kg^−1^ (0.41 mg·kg^−1^ on average) of this element [34]. As shown in Table 2, cadmium (Cd) content was found to be higher as compared to its normal concentration in soil, and was strongly correlated with the distance from the expressway. This would point to the expressway as the source of this element. The authors Turer and Maynard [35], Li [28], and Kluge and Wessolek [36] documented that heavy metal contents in roadside soils decrease with further distance to the road as well as soil depth. In the case of Cd, our studies revealed higher average concentrations at a distance of 0.5 m than at distances of 4.5 m and 25 m from the road. Moreover, the highest concentrations of cadmium (2.26 mg·kg^−1^) in the analyzed soil samples were noted at a depth of 0.00 m–0.10 m; a reduction of approximately 19% occurred at 0.90 m–1.00 m at a sampling location 4.5 m from the road.

A positive correlation, at a *p* < 0.01 level of significance, was observed between Cd and Cu content, whereas the correlation between the content of the analyzed element and the depth at which the samples were collected at a distance of 0.5 m from the road was found to be negative (Table 2).

Lead (Pb) is a highly toxic element to humans, with urban areas characterized by a higher contamination. Road transport has been a major source for lead emissions compared to other anthropogenic factors [37]. The Regulation of the Polish Minister of the Environment [32] sets the permissible level of this trace element in the surface layers of soil at no more than 50 mg·kg^−1^ of soil (protected areas), 100 mg·kg^−1^ of soil (unprotected areas, including farmland) or 600 mg·kg^−1^ of soil (land under transportation routes). Lead is found on roads as a result of vehicle emissions from the combustion of gasoline containing tetraethyl lead [38,39]. Fuels and vehicle emissions are the main sources of this element in roadside soils, which contained from 22.81 mg·kg^−1^ to 92.80 mg·kg^−1^ in samples analyzed by Liu *et al.* [40]. The normal content of Pb in soil in Poland is between 10 and 70 mg·kg^−1^ [34]. The average content of lead in soil samples taken from various sites located at different distances from the road varied from 55.92 (0.5 m) to 22.81 mg·kg^−1^ (25 m), and did not exceed the Polish average (Figure 4). The highest lead content was observed at a distance of 4.5 m from the expressway. Viard *et al.* [41] detected traffic-related Pb contamination at a distance of 320 m from the road. The correlation analysis showed that the concentrations of lead in soil samples at 4.5 m from the road were significantly correlated with those of Zn, Ni and Cu (Table 2).

**Table 2:** The results of the Pearson correlation analysis between the different measured indicators, at five soil depths and three distances from the expressway.

**Section A**							
**Correlation**	**pH**	**EC ^a^**	**Cd**	**Pb**	**Zn**	**Ni**	**Cu**
**EC ^a^**	0.450 *						
**Cd**	−0.178	0.047					
**Pb**	0.292	0.343	0.419 *				
**Zn**	0.239	−0.361	0.730	0.523 *			
**Ni**	0.455 *	−0.042	0.449 *	0.431 *	0.429 *		
**Cu**	0.337	−0.089	0.649 **	0.569 **	0.655 **	0.900 **	
**Depth**	−0.385	0.419 *	−0.638 **	−0.590 **	−0.910 **	−0.434	−0.643 **
**Section B**							
**EC ^a^**	−0.190						
**Cd**	−0.258	0.084					
**Pb**	−0.144	0.832 **	−0.049				
**Zn**	−0.329	0.889 **	0.159	0.857 **			
**Ni**	−0.178	0.848 **	−0.006	0.932 **	0.921 **		
**Cu**	−0.232	0.823 **	0.267	0.846 **	0.927 **	0.945 **	
**Depth**	0.297	−0.788 **	−0.316	−0.707 **	−0.875 **	−0.751 **	−0.803 **
**Section C**							
**EC ^a^**	0.536 *						
**Cd**	−0.209	0.198					
**Pb**	−0.449	−0.229	0.453 *				
**Zn**	−0.865 **	−0.384	0.487 *	0.548 *			
**Ni**	0.209	0.445 *	0.010	0.312	0.045		
**Cu**	−0.864 **	−0.461 *	0.469 *	0.571 **	0.929 **	−0.018	
**Depth**	0.927 **	0.405	−0.148	−0.361	−0.803 **	0.252	−0.252

**^a^** EC = electrical conductivity; * Correlation is significant at the 0.05 level (two-tailed); ** Correlation is significant at the 0.01 level (two-tailed).

A review of literature showed the main sources of zinc (Zn) near roads to be tire wear, corrosion, and oil and cooling liquid leakage [42]. Zn was the only significant element found in tire dust, accounting for 0.02%–0.06% of the PM10 [43]. The share of zinc in the total amount of harmful substances emitted by the transport infrastructure is significant, which, along with the element’s ability to migrate, may pose a danger to the soil-water environment. Zinc can often be found in contaminated soils along with lead and cadmium. Other authors showed that easily mobilised Cd and Zn can be transferred to deeper soil layers [44]. This element most frequently occurs in compounds, and in the case of organic forms is easily absorbed by plants. The contents of zinc in soil samples have been presented in Figure 4. Of the analyzed heavy metals, this element reached the highest levels in the analyzed soil, with concentrations near the road (0.5 m) ranging from 178.25 to 266.41 mg·kg^−1^, which is higher than the national averages in Poland, where the natural average content of zinc in soil is reported to be lower than 50 mg·kg^−1^ of soil. Anthropogenic metals are generally more mobile, and origins are difficult to distinguish; in this study they could be from traffic emissions. The highest average zinc content in the soil samples was found at 0.00–0.10 m depth, where it was as high as 215.34 mg·kg^−1^ of soil. Apeagyei *et al.* [45] observed similar correlations with Zn which suggest braking at traffic lights and stop signs lead to both brake and tire wear. The content of this element was reduced by approximately 96% at a distance of 25 m from the road. Significant positive correlations between zinc and copper content in soils were observed at all tested distances from the road (Table 2). The opposite situation held true for Zn content in relation to the depth at which samples were collected, in which case a significant negative correlation at a *p* < 0.01 level of significance occurred at all analyzed distances (0.5, 4.5 and 25 m from the road).

The concentration of nickel (Ni) in soil was strongly connected with the sampling site, the distance from the road and the depth at which it was collected (Figure 4). The contamination of soil with nickel compounds modifies the physicochemical properties of soil. The following forms of Ni can be found in soil containing minerals: pentlandite ((Fe,Ni)_9_S_8_), cohenite ((Fe,Ni)_3_C), awaruite (Ni_3_Fe) and haxonite ((Fe,Ni)23C_6_) [46]. Dudka [47] determined the average Ni content in Polish soils to be 7.4 mg·kg^−1^ of soil, which was exceeded at a number of the analyzed locations in our study.

The average content of nickel in the soil samples taken nearest the surface (0.00–0.10 m depth) at 0.5 m from the expressway fell within 17.46–124.17 mg·kg^−1^ of soil. The highest average content of nickel was determined on nearest to the road (153.3 mg·kg^−1^), may thus be caused by the main source of nickel predominantly originate from the abrasion of tires and brake pads, from petrol residues as well as through road runoff. The strongest positive correlation (at a *p* < 0.01 level of significance) was observed in the case of nickel and copper content at 0.5 m (*r* = 0.900) and 4.5 m (*r* = 0.945) from the road. Positives significant correlations between nickel and lead content in soils were also noted at a distance of 4.5 m from the road.

The sampling location, distance from the road and depth at which samples were collected significantly influenced the copper (Cu) content of the analyzed soils (Figure 4). Brake wear emission, abrasion of tires and road surface are main source of copper [48]. According to Addo *et al.* [49] the presence of Cu originates is derived from the corrosion of metallic parts, in diesel [50], from car components and lubricants [51]. As given in Figure 4 and Table 2, the concentrations of copper were found to be higher as compared to its normal levels in soil, and were strongly correlated with the distance from the expressway. According to the average national levels for Poland, the natural content is 25 mg·kg^−1^ [47], while the global concentration of Cu varies from 11.3 to 107.5 mg·kg^−1^ of soil [52]. The concentration of Cu in the soil samples taken 0.5 m away from expressway varied from 42.96 to 52.70 mg·kg^−1^ of soil with an average value of 47.79 mg·kg^−1^ of soil. This is proof that traffic and the emission of fumes by cars have an impact on the copper content of soil. As can be assumed, the lowest Cu content was observed at a distance of 25 m from the expressway. The analysis of correlation showed that the concentration of copper in soil samples taken 4.5 m away from the road was significantly correlated with that of Pb, Zn and Ni (Table 2).

### 3.2. Contamination Assessment

Based on heavy metals concentration in studied soil samples, a quantitative analysis of soil pollution around Expressway E30 (Warsaw, Poland) was conducted using the *I_geo_* and *PI* indexes. The results of analysis showed that the mean of *I_geo_* descended in the order of Cu (−0.28) < Zn (0.43) < Ni (0.58) < Cd (1.06) < Pb (1.14). The calculated values and classes for five studied heavy metals for each Section (A, B and C) are listed in Table 3. The *I_geo_* values revealed that in Section A (0.5 m from the edge of the road) the value for Pb, Cd, Ni and Cu metals fell into class 2 (moderately contaminated), and for Zn into class 3 (moderately to strongly contaminated). The *I_geo_* values for Sections B and C (4.5 m and 25 m from the edge of the road, respectively) for most heavy metals fell in classes 0 and 1 (uncontaminated and uncontaminated to moderately contaminated, respectively) except for Pb and Cd in Section B, class 2 (moderately contaminated). The analysis of Pollution Index (*PI*) showed that the mean values were in the order of Cu (1.90) < Ni (2.59) < Cd (3.25) < Zn (3.52) < Pb (3.91). The *PI* values of all analyzed heavy metals in Section A ranged from 3.57 to 7.68 indicating that the soil was highly contaminated; in Section B from 2.04 to 3.77 (middle and high contamination level); in Section C form 0.50 to 2.59 (low and middle contamination level).

**Table 3:** Geo-accumulation index and pollution index values of analyzed metals in topsoil from Warsaw Expressway (E30).

Section	Zn	Pb	Cd	Ni	Cu	Zn	Pb	Cd	Ni	Cu
	*I_geo_* mean values (*n* = 6) for topsoil (0.00–0.30 m)					*I_geo_* class				
A	2.33	1.89	1.24	1.34	1.15	3	2	2	2	2
B	0.52	1.14	1.20	0.41	−0.35	1	2	2	1	0
C	−1.57	0.40	0.74	−0.02	−1.65	0	1	1	0	0
Mean	0.43	1.14	1.06	0.58	−0.28	1	2	2	1	0
	*PI* mean values (*n* = 6) for topsoil (0.00–0.30 m)					*PI* class				
A	7.68	5.69	3.57	4.22	3.85	High	High	High	High	High
B	2.35	3.77	3.57	2.04	1.33	Middle	High	High	Middle	Middle
C	0.53	2.28	2.59	1.50	0.50	Low	Middle	Middle	Middle	Low
Mean	3.52	3.91	3.25	2.59	1.90	High	High	High	Middle	Middle

This analysis revealed that the soil collected along an Expressway (E30) in an ecologically valuable area (central Poland) contained high contamination due to higher concentrations of zinc (Zn), lead (Pb) and cadmium (Cd).

## 4. Conclusions

Heavy metals derived from the transportation infrastructure may have a negative influence on the individual elements of the natural environment. The chemical composition of soils depends on the distance from the road, depth and location. Soils found directly next to the analyzed stretch of road were characterized by the highest values of pH and EC. The highest values of the analyzed heavy metals were found in soil samples collected at a distance nearest to the road (0.5 m), and their concentration was shown to decrease as the distance from the expressway increased. The contents of Cd, Cu and Zn determined in the present study were higher than the Polish national averages and strongly correlated with the distance from the expressway. In the case of Ni and Pb, the average values were not exceeded. The contents of all analyzed heavy metals decreased in soil samples taken from deeper layers of the soil at all distances from the road.

The calculated results of geo-accumulation and pollution indexes of heavy metals revealed that the order of *I_geo_* and *PI* are as following: Cu < Zn < Ni < Cd < Pb and Cu < Ni < Cd < Zn < Pb. The high *I_geo_* values for Zn, Pb and Cd in soils may be caused by road traffic activities. The assessment results of *PI* also support that Zn, Pb, Cd, Ni and Cu levels in the topsoil layer present high pollution.
